# Cooperative Interaction between the MUC1-C Oncoprotein and the Rab31 GTPase in Estrogen Receptor-Positive Breast Cancer Cells

**DOI:** 10.1371/journal.pone.0039432

**Published:** 2012-07-09

**Authors:** Caining Jin, Hasan Rajabi, Sean Pitroda, Ailing Li, Akriti Kharbanda, Ralph Weichselbaum, Donald Kufe

**Affiliations:** 1 Department of Medical Oncology, Dana-Farber Cancer Institute, Harvard Medical School, Boston, Massachusetts, United States of America; 2 Department of Radiation and Cellular Oncology, University of Chicago, Chicago, Illinois, United States of America; 3 Department of Pathology, Brigham and Women’s Hospital, Harvard Medical School, Boston, Massachusetts, United States of America; University of Edinburgh, United Kingdom

## Abstract

Rab31 is a member of the Ras superfamily of small GTPases that has been linked to poor outcomes in patients with breast cancer. The MUC1-C oncoprotein is aberrantly overexpressed in most human breast cancers and also confers a poor prognosis. The present results demonstrate that MUC1-C induces Rab31 expression in estrogen receptor positive (ER+) breast cancer cells. We show that MUC1-C forms a complex with estrogen receptor α (ERα) on the *Rab31* promoter and activates *Rab31* gene transcription in an estrogen-dependent manner. In turn, Rab31 contributes to the upregulation of MUC1-C abundance in breast cancer cells by attenuating degradation of MUC1-C in lysosomes. Expression of an inactive Rab31(S20N) mutant in nonmalignant breast epithelial cells confirmed that Rab31 regulates MUC1-C expression. The functional significance of the MUC1-C/Rab31 interaction is supported by the demonstration that Rab31 confers the formation of mammospheres by a MUC1-C-dependent mechanism. Analysis of microarray databases further showed that (i) Rab31 is expressed at higher levels in breast cancers as compared to that in normal breast tissues, (ii) MUC1+ and ER+ breast cancers have increased levels of Rab31 expression, and (iii) patients with Rab31-positive breast tumors have a significantly decreased ten-year overall survival as compared to those with Rab31-negative tumors. These findings indicate that MUC1-C and Rab31 function in an autoinductive loop that contributes to overexpression of MUC1-C in breast cancer cells.

## Introduction

Rab proteins are members of the Ras superfamily of small GTPases [1]. The Rab GTPases function in receptor internalization, recycling and signaling [2]. Rab31, a Rab5 subfamily GTPase, is involved in the trafficking of early endosomes [3]. Rab31 blocks insulin-stimulated translocation of the Glut4 glucose transporter from endosomes to the cell membrane [4]. In addition, Rab31 is required for transport of mannose 6-phosphate receptors from the trans-Golgi network to endosomes [5]. Interestingly, high levels of Rab31 expression in tumors from 280 node-negative breast cancer patients were shown to be significantly associated with decreased metastasis-free and overall survival [6]. However, no insights are available regarding a potential functional role for Rab31 in breast cancer.

Mucin 1 (MUC1) is a heterodimeric transmembrane glycoprotein that is overexpressed in about 90% of human breast cancers [7], [8], [9]. MUC1 consists of two subunits that form a complex at the cell membrane after translation and autocleavage of a single polypeptide [7], [9]. The MUC1 N-terminal subunit (MUC1-N) contains glycosylated tandem repeats that are a characteristic of mucin family members. The MUC1 C-terminal subunit (MUC1-C) spans the cell membrane and contains a cytoplasmic domain that interacts with diverse effectors, such as the epidermal growth factor receptor (EGFR) and ErbB2, that have been linked to transformation [7], [9]. Overexpression of MUC1-C, as found in human breast cancers, is sufficient to induce anchorage-independent growth and tumorigenicity [10], [11]. The overexpression of MUC1 in transgenic mouse models is also associated with the induction of breast tumors [12], [13]. MUC1-C is internalized from the cell membrane by clathrin-mediated endocytosis [14] and is then recycled from endosomes back to the cell membrane [15]. The overexpression of MUC1 in breast cancer cells is also associated with accumulation of MUC1-C in the cytoplasm by a mechanism that likely involves retrograde trafficking during endocytosis and movement to the endoplasmic reticulum [7], [9]. Cytosolic MUC1-C interacts with importin-β and is transported to the nucleus [16], where it interacts with estrogen receptor α (ERα) and promotes ERα-mediated gene transcription [17].

The present studies demonstrate that MUC1-C induces Rab31 expression in breast cancer cells. MUC1-C forms a complex with ERα on the *Rab31* promoter and activates *Rab31* gene transcription. In turn, Rab31 contributes to the upregulation of MUC1-C levels in an autoinductive loop. These findings are further supported by the demonstration that Rab31 expression is increased in primary breast cancers that are positive for both ERα and MUC1.

## Results

### MUC1 Upregulates Rab31 Expression

**Figure 1:**
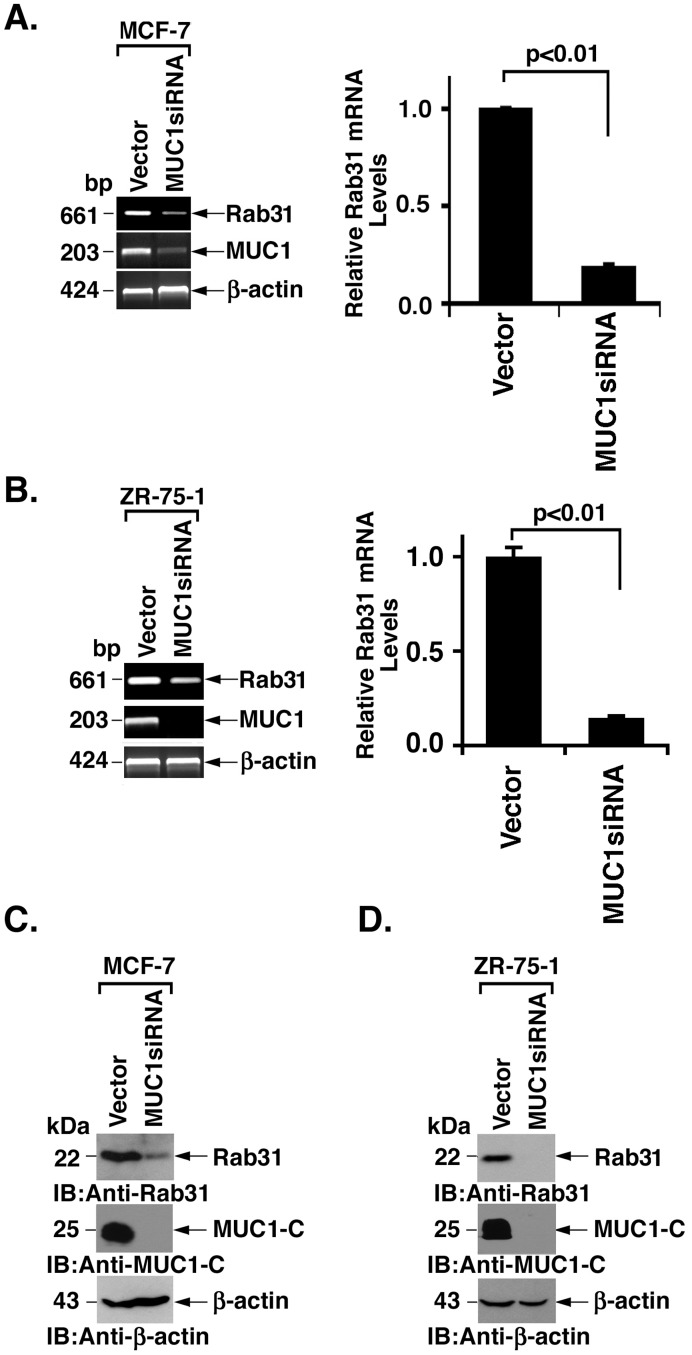
MUC1 induces Rab31 expression. A and B. RNA from the indicated MCF-7 (A) and ZR-75-1 (B) cells was analyzed by RT-PCR using primers designed to detect the indicated transcripts (left). RNA was also analyzed for Rab31 mRNA levels by qRT-PCR (right). The results (mean±SD of three determinations) are expressed as relative Rab31 mRNA levels compared to that obtained in the vector cells. C and D. Lysates from the indicated MCF-7 (C) and ZR-75-1 (D) cells were immunoblotted with the indicated antibodies.

Gene microarray analysis of MCF-7 breast cancer cells demonstrated that silencing of MUC1 is associated with decreases in Rab31 expression. To confirm this observation, MCF-7 cells stably expressing an empty vector (MCF7/vector) or MUC1siRNA (MCF-7/MUC1siRNA) were analyzed for Rab31 mRNA levels by RT-PCR. Downregulation of MUC1-C mRNA levels was associated with a decrease in Rab31 transcripts (Fig. 1A, left). Quantitative RT-PCR confirmed that Rab31 transcripts are significantly lower in MCF-7/MUC1siRNA cells as compared to that in MCF-7/vector cells (Fig. 1A, right). Similar results were obtained when mRNA from ZR-75-1 breast cancer cells was analyzed by RT-PCR (Fig. 1B, left) and qRT-PCR (Fig. 1B, right). In concert with these results, MUC1-C silencing was associated with decreases in the abundance of Rab31 protein in MCF-7 (Fig. 1C) and ZR-75-1 (Fig. 1D) cells. These findings indicate that MUC1-C functions in upregulating Rab31 expression.

### *Rab31* Promoter is Activated by a MUC1-dependent Mechanism

**Figure 2:**
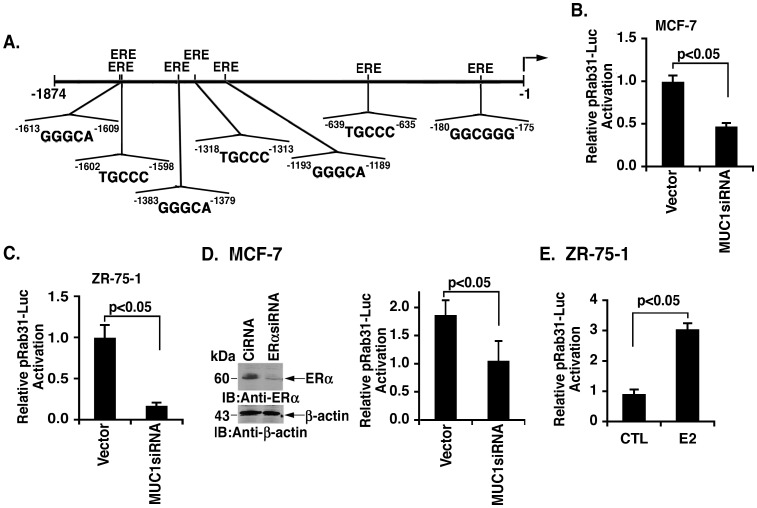
Activation of the *Rab31* promoter by MUC1 and ERα. A. Schematic representation of the *Rab31* promoter with localization of putative estrogen response elements (EREs). B and C. The indicated MCF-7 (B) and ZR-75-1 (C) cells were transfected with pRab31-Luc and *Renilla* plasmids for 48 h and then assayed for luciferase activity. The results are expressed as the relative luciferase activity (mean±SD of three determinations) compared to that obtained in the vector cells. D. MCF-7 cells were transiently transfected with control and ERα siRNA pools. Lysates were immunoblotted with the indicated antibodies (left). The cells were also transfected with pRab31-Luc and *Renilla* plasmids for 48 h. The results are expressed as the relative luciferase activity (mean±SD of three determinations) compared to that obtained in the CsiRNA-transfected cells (right). E. ZR-75-1 cells were transfected with pRab31-Luc and *Renilla* plasmids for 24 h. The cells were then cultured in phenol red-free medium with 2% charcoal dextran-treated serum for 72 h, treated with E2 for 24 h and assayed for luciferase activity. The results are expressed as the relative luciferase activity (mean±SD of three determinations) compared to that obtained in the untreated control (CTL) cells.

To assess the effects of MUC1 on *Rab31* gene transcription, we cloned a 1874 bp region upstream to the *Rab31* transcription start site and inserted those sequences in a luciferase expression vector (pRab31-Luc). Analysis of the *Rab31* promoter region incorporated into pRab31-Luc identified putative consensus binding sites for diverse transcription factors, including multiple potential ERα responsive elements (EREs) (Fig. 2A). Transfection of MCF-7/vector and MCF-7/MUC1siRNA cells with pRab31-Luc demonstrated that silencing MUC1 results in repression of the *Rab31* promoter (Fig. 2B). Similar results were obtained in studies of ZR-75-1 cells, indicating that MUC1-C confers activation of *Rab31* transcription (Fig. 2C). Previous work showed that MUC1-C associates with ERα and promotes activation of ERα target genes [17]. To assess dependence on ERα, we transfected MCF-7 cells with control or ERα siRNA pools to transiently decrease ERα levels (Fig. 2D, left). Silencing of ERα was associated with a decrease in pRab31-Luc activation, consistent with ERα-mediated induction of the *Rab31* promoter (Fig. 2D, right). To provide further support for *Rab31* gene activation by estrogen signaling, ZR-75-1 cells were transfected with pRab31-Luc, cultured in estrogen-depleted medium, and then stimulated with estradiol (E2). Compared to unstimulated cells, E2 treatment was associated with a 3-fold increase in pRab31-Luc activation (Fig. 2E). These findings indicate that *Rab31* promoter is activated by a mechanism dependent on ERα and MUC1-C.

### E2 Induces Rab31 Expression by a MUC1-C-dependent Mechanism

**Figure 3:**
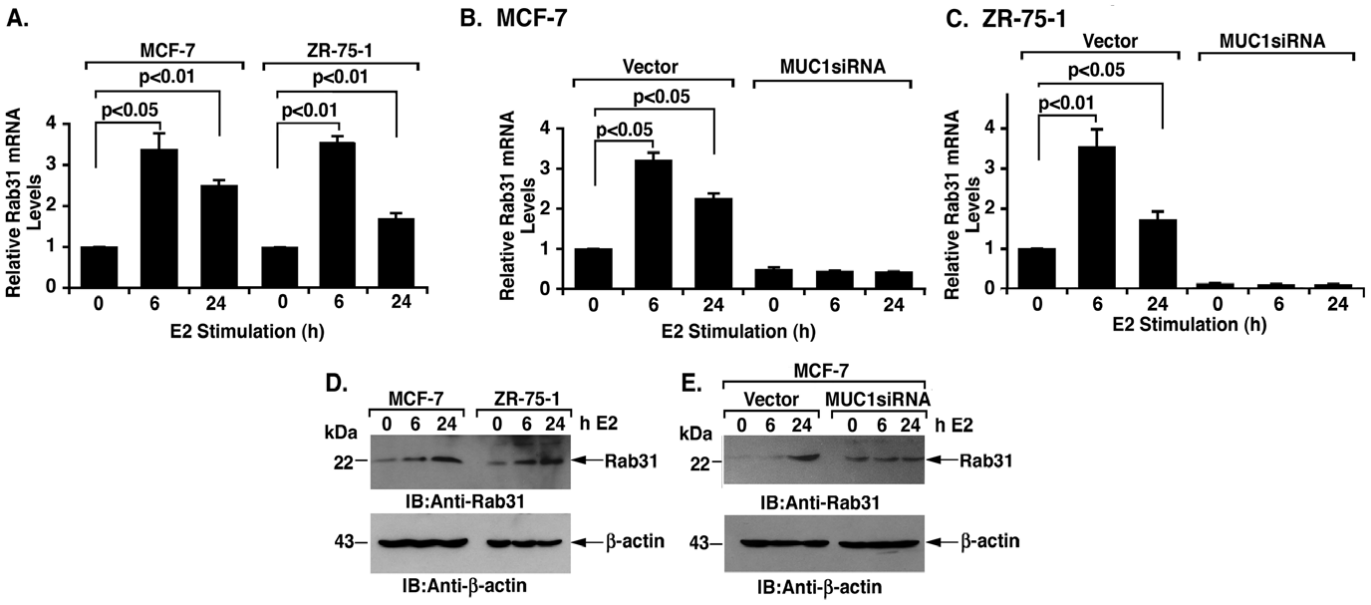
E2-induced Rab31 expression is dependent on MUC1. A. RNA from MCF-7 (left) and ZR-75-1 (right) cells stimulated with E2 for the indicated times was analyzed by qRT-PCR. The results (mean±SD of three determinations) are expressed as relative Rab31 mRNA levels compared to that obtained in unstimulated cells. B and C. RNA from the indicated MCF-7 (B) and ZR-75-1 (C) cells stimulated with E2 for 0, 6 or 24 h was analyzed by qRT-PCR. The results (mean±SD of three determinations) are expressed as relative Rab31 mRNA levels compared to that obtained in the unstimulated vector cells. D. Lysates from MCF-7 (left) and ZR-75-1 (right) cells stimulated with E2 for 0, 6 and 24 h were immunoblotted with the indicated antibodies. E. Lysates from the indicated MCF-7 cells stimulated with E2 for 0, 6 and 24 h were immunoblotted with the indicated antibodies.

In further support for involvement of ERα in Rab31 expression, E2 treatment of MCF-7 cells was associated with a >3-fold increase in Rab31 mRNA levels at 6 h and then a modest decline by 24 h (Fig. 3A, left). Stimulation of ZR-75-1 cells with E2 was also associated with a transient increase in Rab31 mRNA levels at 6 h (Fig. 3A, right). To assess dependence on MUC1-C, E2 treatment studies were performed on MCF-7/vector and MCF-7/MUC1siRNA cells. Stimulation of MCF-7/vector cells with E2 was associated with increases in Rab31 mRNA levels (Fig. 3B, left). By contrast, E2 stimulation had no apparent effect on Rab31 expression in MCF-7/MUC1siRNA cells (Fig. 3B, right). Studies performed with ZR-75-1/vector and ZR-75-1/MUC1siRNA cells further demonstrated that MUC1-C is necessary for E2-induced increases in Rab31 transcripts (Fig. 3C, left and right). Consistent with these results, E2 stimulation of MCF-7 and ZR-75-1 cells was associated with increases in Rab31 abundance (Fig. 3D, left and right). Moreover, this response to E2 was attenuated by silencing MUC1-C (Fig. 3E). These findings indicate that E2 stimulates Rab31 expression at the mRNA and protein levels by a MUC1-C-dependent mechanism.

### MUC1-C Associates with ERα on the *Rab31* Gene Promoter

**Figure 4:**
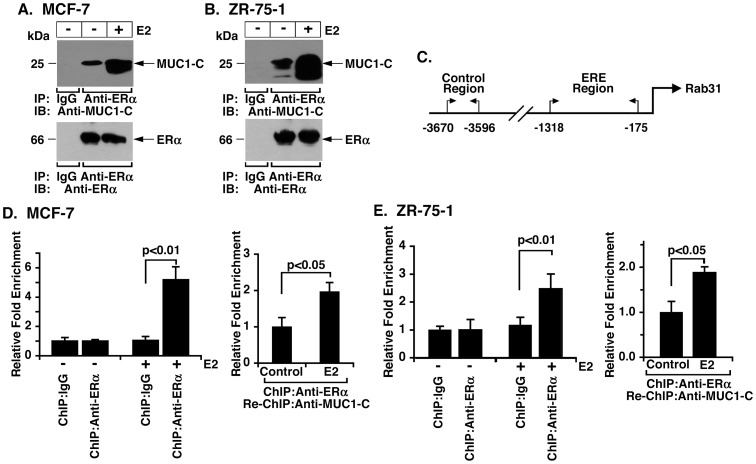
MUC1-C associates with ERα on the *Rab31* promoter. A and B. Lysates from MCF-7 (A) and ZR-75-1 (B) cells left untreated or stimulated with E2 for 24 h were immunoprecipitated with anti-ERα or a control IgG. The precipitates were immunoblotted with anti-MUC1-C or anti-ERα. C. Schema of the *Rab31* promoter highlighting the positions of the control region (CR) and the proximal region encompassing estrogen response elements (EREs). D and E. Soluble chromatin from MCF-7 (D) and ZR-75-1 (E) cells left untreated or stimulated with E2 for 24 h was precipitated with a control IgG or anti-ERα. The precipitates were analyzed for *Rab31* promoter ERE or CR sequences (left). The results (mean±SD of three determinations) are expressed as the relative fold enrichment compared to that obtained with the IgG control. In re-ChIP experiments, the anti-ERα precipitates were released, reimmunoprecipitated with anti-MUC1-C, and then analyzed for *Rab31* promoter sequences (right). The results (mean±SD of three determinations) are expressed as the relative fold enrichment compared to that obtained with the unstimulated control.

Coimmunoprecipitation studies performed on lysates from MCF-7 cells demonstrated that MUC1-C associates with ERα and that this interaction is increased in the response to E2 stimulation (Fig. 4A). Analysis of control and E2-stimulated ZR-75-1 cells demonstrated similar results (Fig. 4B). Chromatin immunoprecipitation (ChIP) studies were then performed on control and E2-stimulated MCF-7 cells to assess the presence of ERα and MUC1-C on the *Rab31* promoter (Fig. 4C). Stimulation with E2 was associated with a ∼5-fold increase in ERα occupancy compared to that obtained with a control IgG (Fig. 4D, left). Re-ChIP studies further demonstrated that MUC1-C is detectable with ERα on the Rab31 promoter (Fig. 4D, right). Analysis of ZR-75-1 cells similarly showed that ER**α** occupancy of the *Rab31* promoter is increased by E2 stimulation (Fig. 4E, left) and that this response is associated with formation of ERα/MUC1-C complexes (Fig. 4E, right). These findings indicate that MUC1-C coactivates *Rab31* gene transcription by interacting with ERα on the *Rab31* promoter.

### Rab31 Upregulates MUC1-C Expression

**Figure 5:**
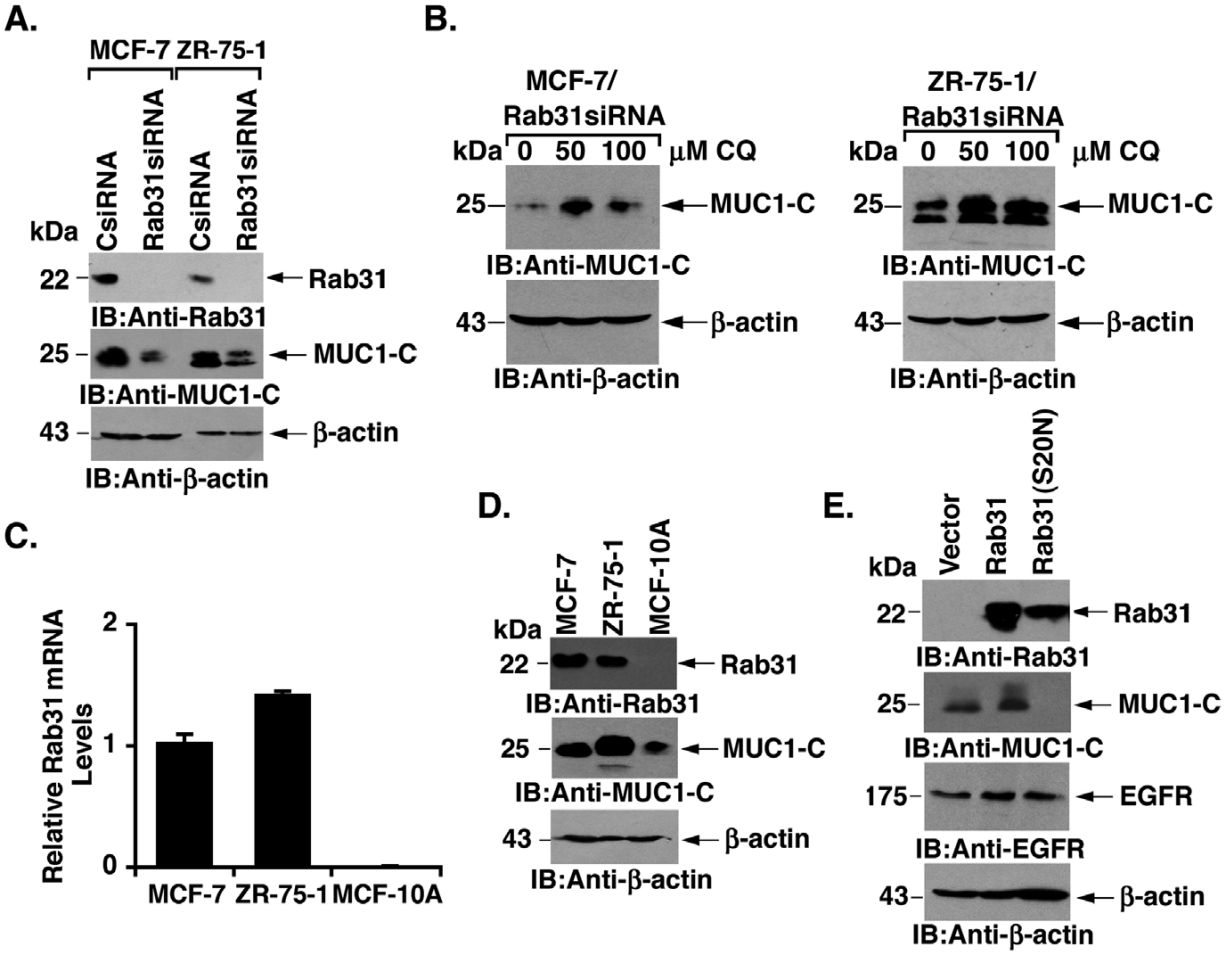
Rab31 upregulates MUC1-C expression. A. MCF-7 (left) and ZR-75-1 (right) cells were stably transfected to express a control siRNA (CsiRNA) or a Rab31 siRNA. Lysates from the transfectants were immunoblotted with the indicated antibodies. B. MCF-7/Rab31siRNA (left) and ZR-75-1/Rab31siRNA (right) cells were treated with the indicated concentrations of chloroquine (CQ) for 24 h. Lysates from the treated cells were immunoblotted with the indicated antibodies. C. RNA from MCF-7, ZR-75-1 and MCF-10A cells was analyzed for Rab31 mRNA levels by qRT-PCR. The results (mean+SD of three determinations) are expressed as relative Rab31 mRNA levels compared to that obtained for MCF-7 cells. D. Lysates from MCF-7, ZR-75-1 and MCF-10A cells were immunoblotted with the indicated antibodies. E. MCF-10A cells were stably transfected to express an empty vector, Rab31 or Rab31(S20N). Lysates from the transfected cells were immunoblotted with the indicated antibodies.

Rab31 functions in the trafficking of proteins, such as MUC1-C, that are processed in early endosomes. To determine whether Rab31 contributes to the regulation of MUC1-C, we silenced Rab31 in MCF-7 cells. Notably, downregulation of Rab31 was associated with a decrease in MUC1-C abundance (Fig. 5A, left). Silencing of Rab31 in ZR-75-1 cells also resulted in a decline in MUC1-C levels (Fig. 5A, right). Analysis of MUC1 transcripts by qRT-PCR demonstrated that silencing Rab31 has no apparent effect on MUC1-C expression at the mRNA level (data not shown). Consequently, studies were performed to determine whether Rab31 regulates processing of MUC1-C from the endosome to the lysosome. Indeed, treatment of MCF-7/Rab31siRNA cells with the lysosome inhibitor chloroquine (CQ) was associated with an increase in MUC1-C abundance (Fig. 5B, left). Similar results were obtained in CQ-treated ZR-75-1/Rab31siRNA cells (Fig. 5B, right). To extend this analysis, we examined Rab31 expression in non-malignant MCF-10A breast epithelial cells. Compared to that in MCF-7 and ZR-75-1 cells, Rab31 transcripts were substantially lower in MCF-10A cells (Fig. 5C). Moreover, Rab31 and MUC1-C protein levels were in lower abundance in MCF-10A cells relative to that in the breast cancer cells (Fig. 5D). We therefore transfected MCF-10A cells to stably express Rab31 or an inactive Rab31(S20N) mutant (Fig. 5E). Expression of Rab31 in MCF-10A cells was associated with a modest increase in MUC1-C levels (Fig. 5E). In contrast, the Rab31(S20N) mutant completely suppressed MUC1-C expression (Fig. 5E). As a control, levels of EGFR protein were unaffected by expression of Rab31 or Rab31(S20N) (Fig. 5E), supporting the selective effects of Rab31 on MUC1-C expression.

### Rab31 Supports Growth of MCF-10A Cells as Mammospheres by a MUC1-C-dependent Mechanism

**Figure 6:**
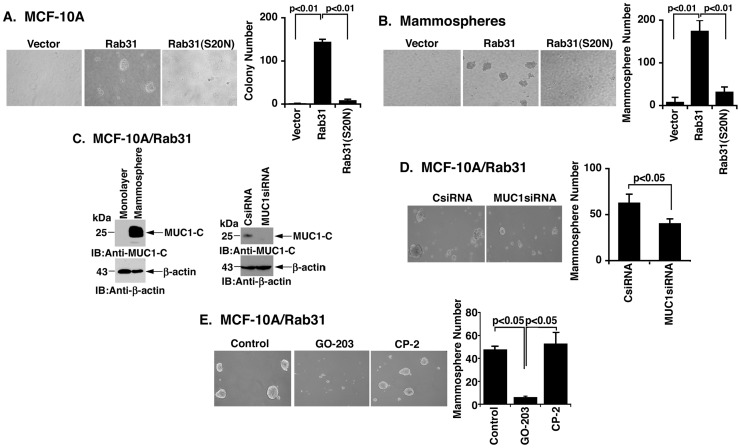
Rab31 promotes MCF-10A mammosphere formation by a MUC1-C-dependent mechanism. A. The indicated MCF-10A cells were seeded in soft agar. After culturing for 3 weeks, photomicrographs were obtained (left) and the numbers of colonies (mean±SD of three replicates) were determined by counting clusters of >20 cells. B. The indicated MCF-10A cells were seeded under conditions for the growth of mammospheres. After culturing for 3 weeks, photomicrographs were obtained (left) and the numbers of mammospheres (mean±SD of three replicates) were determined by counting spheres of >20 cells (right). C. Lysates from MCF-10A/Rab31 cells grown as an adherent monolayer and as mammospheres were immunoblotted with the indicated antibodies (left). MCF-10A/Rab31 cells were transfected with control and MUC1 siRNAs. Lysates were immunoblotted with the indicated antibodies (right). D. MCF-10A/Rab31 cells transfected with the indicated siRNAs were cultured for the growth of mammospheres. After 3 weeks, photomicrographs were obtained (left) and the numbers of mammospheres (mean±SD of three replicates) were determined by counting spheres of >20 cells (right). E. MCF-10A/Rab31 cells were cultured for the growth of mammospheres in the absence (Control) and presence of 5 µM GO-203 or CP-2. After 3 weeks, photomicrographs were obtained (left) and the numbers of mammospheres (mean±SD of three replicates) were determined by counting spheres of >20 cells (right).

To assess affects of Rab31 on growth, the MCF-10A cells were first seeded in soft agar. MCF-10A/vector cells failed to form colonies (Fig. 6A, left). In contrast, MCF-10A/Rab31 cells formed colonies and this response was substantially suppressed with the Rab31(S20N) mutant (Fig. 6A, left and right). Mammosphere structures are formed by culturing tumor-initiating cells under nonadherent and nondifferentiating conditions [18], [19]. In concert with previous observations with parental MCF-10A cells [20], MCF-10A/vector cells failed to form mammospheres (Fig. 6B, left). However, mammospheres were formed with MCF-10A/Rab31 cells (Fig. 6B, left). Moreover, for MCF-10A/Rab31(S20N) cells, small clusters were detectable that did not develop into mammosphere structures (Fig. 6B, left). Quantitation of mammosphere number confirmed dependence of MCF-10A cells on Rab31 for development of these structures (Fig. 6B, right). Immunoblot of MCF-10A/Rab31 cells growing as a monolayer and as mammospheres demonstrated the marked upregulation of MUC1-C levels under the mammosphere culture conditions (Fig. 6C, left). To assess involvement of MUC1-C in mammosphere formation, MUC1-C was silenced in the MCF-10A/Rab31 cells (Fig. 6C, right). Analysis of the MCF-10A/Rab31 cells expressing a control or MUC1 siRNA demonstrated that silencing MUC1-C is associated with a significant decrease in the formation of mammospheres (Fig. 6E, left and right). Using another approach to assess involvement of MUC1-C, we treated the MCF-10A/Rab31 cells with the cell-penetrating peptide GO-203 that blocks MUC1-C function [21], [22]. As a control, cells were also treated with an inactive cell-penetrating peptide CP-2. Treatment with GO-203, but not CP-2, was associated with a significant decrease in mammosphere formation (Figs. 6F, left and right). These findings indicate that Rab31 confers the ability of MCF-10A cells to form mammospheres by a MUC1-C-dependent mechanism.

### Expression of Rab31 and MUC1 in Human Breast Cancers

**Figure 7:**
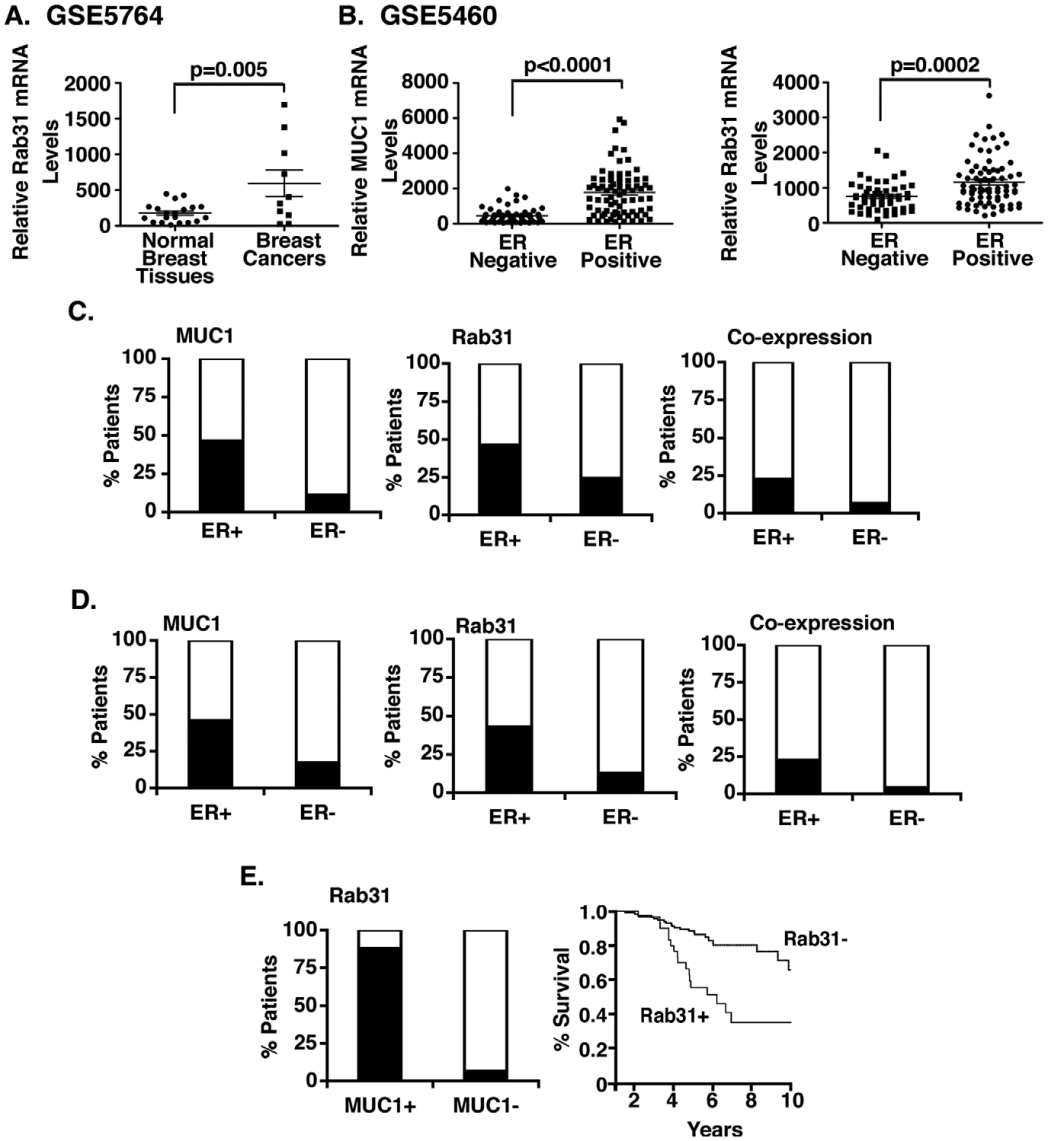
Expression of Rab31 in human breast cancers. A. Analysis of Rab31 mRNA levels in the GSE5764 dataset from 23 normal breast tissues and 10 breast tumors. The results are expressed as the relative Rab31 mRNA levels based on the normalization values in the dataset. B. Analysis of MUC1 mRNA (left) and Rab31 mRNA (right) levels in the GSE5460 dataset from 76 ER+ and 53 ER− breast tumors. Normalized microarray data were separated into ER+ and ER- groups. C and D. Analysis of MUC1 mRNA (left), Rab31 mRNA (middle) and co-expression levels (right) in the Loi dataset (C, 262 ER+ and 45 ER− breast tumors) and van de Vijver dataset (D, 226 ER+ and 69 ER− breast tumors). E. Analysis of Rab31 and MUC1 mRNA co-expression levels in 147 ER+ breast tumors (left). Percentage overall survival for patients with Rab31-positive versus Rab31-negative breast tumors (right; Chanrion dataset).

Analysis of Rab31 expression in the GSE5764 dataset from 20 normal breast tissues and 10 breast tumors demonstrated that Rab31 mRNA levels are significantly higher in breast cancers as compared to normal breast tissue (Fig. 7A). Analysis of the GSE5460 dataset of 76 ER+ and 53 ER− breast tumors further demonstrated that MUC1 expression is significantly higher in ER+ as compared to ER- cancers (Fig. 7B, left). Rab31 expression levels were also significantly higher in ER+ breast cancers (Fig. 7B, right). In the dataset of Loi et al. [23], there are 262 patients with ER+ and 45 patients with ER- breast cancers. MUC1 was expressed in 46.6% (122) of ER+ tumors and 11.1% (5) of ER- tumors (Fisher’s exact test, p<0.0001) (Fig. 7C, left). Rab31 was expressed in 46.2% (121) of ER+ tumors and 24.4% (11) of ER- tumors (Fisher’s exact test, p = 0.0086) (Fig. 7C, middle). Notably, MUC1 and Rab31 were more likely to be co-expressed in ER+ tumors (n = 59, 22.5%) than ER- tumors (n = 3, 6.67%) (Fisher’s exact test, p = 0.015) (Fig. 7C, right). This analysis was expanded to the dataset of van de Vijver et al. [24], in which there are 226 patients with ER+ and 69 patients with ER- breast cancers. MUC1 was expressed in 46.0% (104) of ER+ tumors and 17.4% of ER- tumors (12) (Fisher’s exact test, p<0.0001) (Fig. 7D, left). Rab31 was expressed in 42.9% (97) of ER+ tumors and 13.0% (9) of ER- tumors (Fisher’s exact test, p<0.0001) (Fig. 7D, middle). These results also confirm that MUC1 and Rab31 were more likely to be co-expressed in ER+ tumors (n = 51, 22.6%) than ER- tumors (n = 3, 4.35%) (Fisher’s exact test, p = 0.0003) (Fig. 7D, left). We therefore focused on analysis of the dataset of Chanrion et al. composed of 147 patients with ER+ breast tumors [25]. MUC1 was expressed in tumors from 25 patients (17.0%), while Rab31 was expressed in tumors from 30 patients (20.4%) (Fig. 7E, left). The association between MUC1 and Rab31 expression was highly significant (Fisher’s exact test, p<0.0001) in that MUC1-positive tumors were more likely than MUC1-negative tumors to express Rab31 (n = 22, 88.0% vs. n = 8, 6.56%). Moreover, patients with Rab31-positive tumors had a significantly decreased ten-year overall survival compared with patients with Rab31-negative tumors (35.3% versus 65.9%) (log-rank test, p<0.0001) (Fig. 7E, right).

## Discussion

### MUC1-C Upregulates Rab31 Expression

The overexpression of MUC1 in human breast cancers is associated with targeting of the MUC1-C subunit to the nucleus where it interacts with ERα [7], [9], [17]. The present studies demonstrate that silencing of MUC1-C in ER+ breast cancer cells is associated with downregulation of transcripts encoding the Rab31 GTPase. Little is known about the function of Rab31, other than it is involved in the trafficking of proteins in endosomes [3], [4], [5]. In addition, high levels of Rab31 in tumors from patients with node-negative breast cancers have been associated with decreased survival [6]. However, to our knowledge, there has been no known association between Rab31 and effectors linked to breast cancer. The present findings support a model in which the MUC1-C oncoprotein activates Rab31 expression by an ERα-mediated mechanism. MUC1-C stabilizes ERα by blocking its ubiquitination and degradation [17]. MUC1-C also associates with ERα on estrogen-responsive promoters, enhances ERα occupancy, increases recruitment of p160 coactivators and antagonizes the inhibitory effects of tamoxifen [17]. In the present studies, analysis of the *Rab31* promoter identified potential ERα binding sites and, in ChIP assays, occupancy by ERα. Silencing of ERα suppressed activation of a Rab31 promoter-reporter and decreased Rab31 mRNA levels, indicating that *Rab31* gene transcription is driven by ERα signaling. To our knowledge, Rab31 expression had not been previously linked to ERα-mediated transactivation and this finding provided the basis for assessing the potential involvement of MUC1-C. Indeed, in support of the regulation of Rab31 by MUC1-C, complexes of ERα and MUC1-C were detectable on the *Rab31* promoter. Moreover, silencing MUC1-C suppressed (i) *Rab31* promoter activation, (ii) Rab31 mRNA levels, and (iii) E2-induced Rab31 expression. There had previously been no available information on how the *Rab31* gene is regulated in breast cancer cells, particularly those that express high Rab31 levels. Our results support a model in which the *Rab31* gene is activated by both MUC1-C and ERα in breast cancer cells. These findings, however, do not exclude the possibility that MUC1-C could also contribute to Rab31 expression by ERα-independent mechanisms.

### Rab31 Cooperates with MUC1-C in a Potential Autoinductive Loop

Expression of the MUC1-C subunit at the cell membrane is regulated by clathrin-mediated endocytosis [14], [15]. The role of Rab31 in the trafficking of certain proteins in endosomes invoked the possibility that MUC1-C-mediated induction of Rab31 expression could, in turn, affect MUC1-C levels. Indeed, silencing of Rab31 in breast cancer cells was associated with a decrease in MUC1-C protein, but not transcripts, indicating that Rab31 might prevent processing of endosomal MUC1-C for degradation in lysosomes. In concert with such a model, chloroquine treatment of breast cancer cells silenced for Rab31 was associated with increases in MUC1-C abundance, indicating that Rab31 promotes recycling of MUC1-C, as opposed to its lysosomal degradation. The interaction between Rab31 and MUC1-C was further supported by studies in non-malignant MCF-10A breast epithelial cells, which express low levels of Rab31. Introduction of Rab31 in MCF-10A cells was associated with a modest increase in MUC1-C levels. Strikingly, expression of an inactive Rab31(S20N) mutant resulted in complete suppression of MUC1-C abundance, a response that was selective for MUC1-C in that Rab31(S20N) had no effect on EGFR levels. Rab31, but not Rab31(S20N), also conferred the ability of MCF-10A cells to form mammospheres. Under these nonadherent and nondifferentiating conditions of culturing the MCF-10A/Rab31 cells, the formation of mammospheres was decreased by silencing MUC1-C expression. In addition, treatment of the MCF-10A/Rab31 cells with GO-203, an inhibitor of MUC1-C function, blocked mammosphere formation. These results thus provide support for a cooperative interaction between MUC1-C and Rab31. MUC1-C activates Rab31 expression and, in turn, Rab31 increases MUC1-C levels in a positive feedback autoinductive loop. In this capacity, the functional significance of such a loop is that Rab31 alters the growth characteristics of breast epithelial cells, at least in part, by a MUC1-C-dependent mechanism.

### Association of MUC1 and Rab31 Expression in ER+ Breast Cancers

An experimental model of MUC1-C-induced transformation identified the activation of gene families involved in oncogenesis [26]. Application of MUC1-C-induced genes associated with tumorigenesis to breast cancer databases predicted significant decreases in disease-free and overall survival [26]. Rab31 expression in breast cancers is also significantly associated with decreases in overall survival [6]. The present results demonstrate that Rab31 expression is significantly higher in breast tumors as compared to normal breast tissue. In addition and in concert with our finding that ERα activates *Rab31* gene transcription, Rab31 expression was also significantly higher in ER+ as compared to ER- breast cancers. As noted above, Rab31 expression is not restricted to ER+ breast cancer cells, indicating that ERα-independent mechanisms can also contribute to *Rab31* gene transcription. Our results further demonstrate that Rab31 and MUC1 are significantly co-expressed in ER+ breast cancers. These findings are thus consistent with a model in which MUC1-C coactivates ERα-mediated *Rab31* transcription in breast cancer cells growing in vitro and as primary tumors. Other studies have demonstrated that MUC1-C induces genes involved in cholesterol and fatty acid metabolism [27]. The MUC1-C-induced gene set associated with lipid metabolism was applied to two independent databases from patients with ER+ breast tumors who were treated with tamoxifen. The results showed that patients with tumors expressing MUC1 and the lipid metabolic pathways are at higher risk for recurrence and death [27]. A positive correlation was also found between the MUC1-C-induced gene set and the ER signaling pathway [27]. These findings indicate that the autoinductive loop between MUC1-C and Rab31 found in ER+ breast cancer cells in the present studies could contribute, at least in part, to ER+ breast tumors that fail to respond to tamoxifen treatment. A direct inhibitor of MUC1-C is presently under study in a Phase I trial for patients with refractory solid tumors. Based on the present findings, patients with breast cancer that co-express MUC1-C and Rab31, and perhaps are resistant to tamoxifen, could be candidates for the targeting of MUC1-C with this agent.

## Materials and Methods

### Cell Culture

Human MCF-7 breast cancer cells were cultured in Dulbecco’s modified Eagle’s medium with 10% heat-inactivated fetal bovine serum (FBS), 100 U/ml penicillin, 100 µg/ml streptomycin and 2 mM L-glutamine. Human ZR-75-1 cells were grown in RPMI-1640 medium (ATCC) with 10% FBS, antibiotics and L-glutamine. Human MCF-10A mammary epithelial cells were grown in mammary epithelial growth medium (MEGM, Lonza). In certain experiments, cells were cultured in phenol red-free MEM medium containing 2% charcoal dextran-treated calf serum and then treated with 10 nM estradiol (E2; Sigma). Cells were also treated with chloroquine (CQ; Sigma). Stable silencing of MUC1 or Rab31 was performed by transduction of cells with a lentivirus expressing Mission shRNAs (Sigma) and selection in puromycin. For ERα silencing, cells were transfected with smart pool ERα siRNAs (Dharmacon).

### RT-PCR and qRT-PCR

Total RNA was isolated from cells using an RNeasy Mini kit (Qiagen). cDNAs were synthesized with 0.3–1 µg RNA using the first-strand cDNA synthesis kit (Invitrogen). Expression of Rab31, MUC1 and β-actin was analyzed with 1 µl of cDNA using Taq DNA polymerase (Promega). For quantitative PCR, the SYBR green qPCR assay kit (Applied Biosystems) was used with 5 µl of 20-fold diluted cDNA from each sample, and the samples were amplified with the ABI Prism 7300 machine (Applied Biosystems). Primers used for RT-PCR and qRT-PCR are listed in Tables S1 and S2, respectively.

### Immunoprecipitation and Immunoblot Analysis

Cell lysates were incubated with control IgG or anti-ERα (Santa Cruz Biotechnology) overnight at 4°C. Protein G-Sepharose beads (GE Health Care Life Sciences) were added for another 2 h. The immunoprecipitates and lysates not subjected to precipitation were immunoblotted with anti-Rab31 (C-15; Santa Cruz Biotechnology), anti-MUC1-C (Ab5; Neomarkers), anti-ERα, anti-EGFR (Santa Cruz Biotechnology) and anti-β-actin (Sigma). Immune complexes were detected with horseradish peroxidase-conjugated secondary antibodies and enhanced chemiluminescence (Amersham Biosciences).

### Rab31 Promoter-reporter Assays

The *Rab31* promoter region from −1 to −1874 was cloned into the pGL3 luciferase vector (Promega). Control pGL3 or pRab31-Luc constructs were transfected with the *Renilla* plasmid into cells in the presence of Lipofectamine. At 48 h after transfection, cells were lysed, and luciferase reporter activity was measured using the Promega Dual Glo kit.

### Chromatin Immunoprecipitation (ChIP) Assays

Soluble chromatin was prepared from 2–3×10^6^ cells as described [28] and precipitated with anti-ERα or a control nonimmune IgG. For re-ChIP assays, complexes from the initial ChIP were eluted and reimmunoprecipitated with anti-MUC1-C as described [28]. For PCR, 2 µl from a 50 µl DNA sample was used with the indicated primers (Table S3) and 25–35 cycles of amplification. Fold enrichment was calculated as described [28].

### Site-directed Mutagenesis

The Rab31(S20N) mutant was generated using the Quikchange XLII Site-Directed Mutagenesis kit (Stratagene).

### Colony Formation in Soft Agar

Cells (3×10^4^) were suspended in 2 ml of 0.35% (wt/vol) agar containing DMEM/10% fetal bovine serum and overlaid onto a 0.75% (wt/vol) agar solution in 6-well plates. One ml of fresh medium was added once a week. Colonies >20 cells were counted and imaged after 3 weeks of incubation.

### Mammosphere Formation

Cells (4×10^4^) in 2 ml serum-free DMEM/F12 (Invitrogen) media, supplemented with B27 (1∶50, Invitrogen), 0.4% BSA, 20 ng/ml EGF, and 4 µg/ml insulin (Sigma) were seeded in 6-well ultra-low adherent plates (StemCell Technologies). At 3 weeks, spheres containing >20 cells were photographed and counted.

### Analysis of Breast Cancer Datasets

Five publicly available datasets were analyzed that contain normal or breast tumor expressional data from 30 [29], 129 [30], 307 [23], 295 [24], and 147 [25] patients. All statistical analyses were performed using JMP 9.0 (SAS Institute Inc., Cary, NC, USA). The raw signal intensity for each probe set ID of interest for each patient was normalized to the average value of the probe set ID across all patients. Multiple probe set IDs for a given gene were averaged for each patient sample to obtain a representative expression value for each gene. MUC1 and Rab31 expression were defined as having a normalized expression value greater than one. Fisher’s exact test was used to determine differences in association between two groups. Survival analysis was performed using Kaplan-Meier statistics with log-rank tests to test the null hypothesis of no difference in survival functions between patient groups.

## Supporting Information

Table S1 **Primers used for RT-PCR of Rab31 and MUC1.**
(RTF)

Table S2 **Primers used for qRT-PCR of Rab31.**
(RTF)

Table S3 **Primers used in ChIP assays of Rab31 promoter.**
(RTF)
