# Supplementary material for: Cooperative Interaction between the MUC1-C Oncoprotein and the Rab31 GTPase in Estrogen Receptor-Positive Breast Cancer Cells
Source: PLoS One. 2012 Jul 9;7(7):e39432. doi: 10.1371/journal.pone.0039432 (PMC3392244; doi:10.1371/journal.pone.0039432)
Supplement: Table S1 — Primers used for RT-PCR of Rab31 and MUC1. (RTF) [file pone.0039432.s001.rtf]

Supplemental Table S1. Primers used for RT-PCR of Rab31 and MUC1 

MUC1	 Fwd:  5'- GAAAGAACTACGGGCAGCTGG-3'	
MUC1	 Rev:  5'- CAAGTTGGCAGAAGTGGCTGC-3'	
Rab31	 Fwd:  5'-CGAGCACATGATGGCGATACG-3'	
Rab31	 Rev:  5'-GTCCTTCAGCAGTGCACAGGA-3'	
β-actin	 Fwd:  5'-ATGGATGATGATATCGCCGCGCTC-3'	
β-actin	 Rev:  5'-GGGATAGCACAGCCTGGATA-3'	
