# Supplementary material for: Cooperative Interaction between the MUC1-C Oncoprotein and the Rab31 GTPase in Estrogen Receptor-Positive Breast Cancer Cells
Source: PLoS One. 2012 Jul 9;7(7):e39432. doi: 10.1371/journal.pone.0039432 (PMC3392244; doi:10.1371/journal.pone.0039432)
Supplement: Table S2 — Primers used for qRT-PCR of Rab31. (RTF) [file pone.0039432.s002.rtf]

Supplemental Table S2. Primers used for qRT-PCR of Rab31

Rab31	 Fwd:  5'-TCAGCTGCAGCTGTTATCGT-3'	
Rab31	 Rev:  5'-CTGGACCATGTTCTTTCAGC-3'	
HPRT	 Fwd:  5'-GAAGAGCTATTGTAATGACCAGTC-3'	
HPRT	 Rev:  5'-CAAGCTTGCGACCTTGACCATC-3'	
 
