# Supplementary material for: Cooperative Interaction between the MUC1-C Oncoprotein and the Rab31 GTPase in Estrogen Receptor-Positive Breast Cancer Cells
Source: PLoS One. 2012 Jul 9;7(7):e39432. doi: 10.1371/journal.pone.0039432 (PMC3392244; doi:10.1371/journal.pone.0039432)
Supplement: Table S3 — Primers used in ChIP assays of Rab31 promoter. (RTF) [file pone.0039432.s003.rtf]

Supplemental Table S3. Primers used in ChIP assays of Rab31 promoter

ChIP qPCR ERα primer pair GPH1006171(-)01A	
ChIP qPCR CR region primer pair:
Fwd:  5'-CACCTGACACCAATCCTTTGTG–3'
Rev:  5'-CCAGAACAAGTAGACAGCTCTC–3'	
